# Inequalities in health care utilization for common illnesses among under five children in Bangladesh

**DOI:** 10.1186/s12887-020-02109-6

**Published:** 2020-05-04

**Authors:** Moriam khanam, Emran Hasan

**Affiliations:** 1 Institute of Health Economics, University of Dhaka, Dhaka, 1000 Bangladesh; 2 Department of Economics, Bangladesh University of Professionals (BUP), Dhaka, Bangladesh

**Keywords:** Childhood illnesses, Inequalities, Medical treatment, Utilization, Bangladesh

## Abstract

**Background:**

Reducing child mortality and morbidity is a public health concern globally. Like many other developing countries, Bangladesh is struggling to improve child health status as the use of medical treatment is still not at a satisfactory level. Hence, the objective of this study is to identify the contributing factors for inequalities in the use of medical treatment for common childhood illnesses in Bangladesh.

**Methods:**

The study used data from the latest Bangladesh Demographic and Health Survey (BDHS)-2014. Children who had diarrhea, fever and cough in the 2 weeks preceding the survey were included in this study. Bivariate and multivariate analyses were conducted to unearth the influential factors for medical treatment use among under-five children with childhood illnesses. In the multivariate logistic regression, adjusted odds ratios with *p* values less than 0.05 were considered for determining significant predictors.

**Results:**

This study found that only 37% of children suffering from fever/cough sought medical treatment while this figure was approximately 36% for diarrhea. Age of children, household wealth status, father’s education level, region of residence, number of children in the household, access to electronic media were identified as factors contributing to inequality in health care utilization for common childhood illnesses in Bangladesh.

**Conclusions:**

Various socio-economic factors substantially influence the utilization of medical treatment for childhood illnesses. Therefore, to enhance equitable access to health care for children, interventions should be designed targeting children from households with low socio-economic status. Various awareness-raising health education programs, poverty alleviation programs especially for rural areas can contribute in this regard.

## Background

Globally, child mortality has dropped from 12.6 million in 1990 to 5.3 million in 2017 [1]. Despite such remarkable progress in reducing under 5 mortality rates, disparities exist across countries and regions as 52% deaths of under 5 children took place in sub-Saharan Africa while another 29% child deaths occurred in Central and Southern Asia in 2018 [2]. These deaths across the world are mainly attributed to various infectious diseases that are largely amenable to available preventive treatment and measures [3]. Acute diarrheal diseases and acute respiratory infections are considered as the most common forms of acute childhood illnesses [4]. The World Health Organization (WHO) also estimated that using quick and proper care during episodes of acute diarrheal diseases and acute respiratory infections could reduce child deaths by about 30%. However, the morbidities of children caused by various infectious diseases lead to a huge burden both from health and economic perspectives. One of the main reasons for the slow progress of developing countries in reducing child mortality is the socio-economic inequalities in these countries. Socio-economic inequities – as considered one of the fundamental reasons, are solely responsible for slow progress in terms of reducing child mortality especially in the context of developing countries [4]. Reducing mortality and morbidity of children is critically related to seeking appropriate care from medically trained providers coupled with sufficiently equipped health care facilities [5]. Inefficiency and insufficiency of health care facilities especially for children is prevalent in developing countries. Studies from different countries suggest that health care seeking is not adequate in cases of childhood illnesses [4, 6, 7]. Mothers or caregivers more often resort to over-the-counter drug peddlers, traditional or faith healers, or self-medication [4].

Bangladesh has made noteworthy progress in achieving MDG goal of reducing child mortality, the child mortality rate has dropped from 143.8 per 1000 live births in 1990 to 32.4 per 1000 live births in 2017 [8]. In 2016, the death rate of children under 5 years was 5.5 (per 1000 live births) due to pneumonia while this figure was 2.3 for diarrhea [9]. The country with a view to alleviating child mortality has made an extensive infrastructure development of public healthcare facilities from the local level (ward level) to the national level, where almost all types of treatment are intended to be available to patients typically free of charge [10]. According to Bangladesh Health Facility Survey 2014, 93.6% of the rural health facilities have available outpatient curative care services for sick children while 81.6% of urban health facilities have curative care for children. In Sylhet division, 99.8% health facilities provide health care for children followed by Khulna (98.6%), Barisal (96%), Rangpur (95.5%), Chittagong (92.1%), Dhaka (90%), and Rajshahi (85%) [11]. Evidences show that though health services are supposed to be free at government facilities, patients have to pay the costs of medicines and laboratory tests along with some additional costs [12]. Therefore, the care-seeking behavior for sick under 5 children is significantly low and still the childhood mortality and morbidity rate is sufficiently high in Bangladesh [13]. This poor use of health care services poses a great impediment to attaining child health development mainly in respect of the Sustainable Development Goal (SDG) of ending preventable deaths of under-five by 2030 [14]. One possible reason for this may be the low utilization of health care services from qualified sources for under-five children.

Improving child health status through formulating and implementing proper initiatives, policymakers must have a good understanding of the overall health services utilization. In this regard, an important question to ask is, what the care seeking pattern is and what factors are influencing the utilization of health care. Many studies have been conducted in different countries to identify the care seeking pattern and influential factors for the utilization of health care services in cases of childhood illnesses [4, 12–17]. Wealth possession of households is widely recognized as predictor of health care utilization where children from wealthy families can afford to receive health care [12–14, 16, 18–21]. Besides, parent’s education level, religion were also found to be associated with the inequality in the utilization of child health services [12, 21]. People with higher education have greater perceived benefits from treatment compared to the people with low education [12]. Population from Protestant and Muslim religion are found to have higher likelihood of medical treatment seeking behavior than respondents from Orthodox Christian group [21]. Furthermore, children’s age and sex, mothers’ age, and access to electronic media tend to influence the treatment seeking behavior for childhood illnesses [12, 19–21]. Female children are less likely to receive medical care compared to male children [12]. Another study found that medical care utilization rate was lower among rural children compared to urban children [20]. Some studies mentioned that distance to the health facility reduces the likelihood of utilization of medical care [20, 22].

However, there are few studies in Bangladesh which examined the influence of socio-demographic, household characteristics and geographical access in seeking health care for childhood illnesses on a national scale. Sarker et al. (2016) studied the prevalence of childhood diarrheal disease and their treatment seeking behavior in Bangladesh. They found some of the socio-economic factors, especially, wealth index and number of children in the household as the most important predictors. Another study on prevalence and determinants of acute respiratory tract infections (ARI) of children in Bangladesh found sex of children and wealth status of household as significant determinants of health care seeking [18]. These studies focused on a single disease e.g. diarrhea and ARI. Besides, a study by Mahumud et al. (2019) attempted to explore the inequality of childhood morbidity in Bangladesh. However, the study did not capture the inequality in treatment use [23]. Contingent upon this backdrop, the objective of this study is to find out the determining factors which influence equitable health care utilization among children with fever, cough, and diarrhea in Bangladesh so as to provide information for future planning, policy making, and appropriate interventions in reducing the level of under-five morbidity and mortality in Bangladesh.

## Methods

### Data

This study used data from the latest Bangladesh Demographic and Health Survey (BDHS) 2014, which is a nationally representative cross-sectional household survey. The survey was designed to collect data on demographic characteristics and health indicators. The detailed method of data collection, sampling technique, instruments, survey design, data validity, reliability, and quality control have been described elsewhere [24]. The BDHS Data was collected from June 28, 2014, to November 9, 2014, which covers all the administrative divisions of Bangladesh. Ever-married women who were in the age group of 15–49 years were interviewed. A total of 17,863 women were interviewed in this survey with a 98% response rate. This dataset contains information on child health, reproductive health, and nutritional status of mother and children. BDHS followed standardized procedures for data collection. According to the BDHS report, consent was taken from mothers on behalf of their children who are enrolled in the survey.

The BDHS data set is available publicly online for researchers. However, to use this data for this study, approval was required from and given by MEASURE DHS (Measure Demographic and Health Survey) program office.

### Outcome variable

Children with diarrhea, fever/cough were identified in the DHS data through their mothers’ reporting whether the child had diarrhea and/or fever, and/or cough within the 2 weeks before the survey conducted. The outcome variables were ‘mother sought medical care for her child with fever/cough’ and ‘mother sought medical care for her child with diarrhea’. The outcome variables were coded as “1″ if medical care was sought and “0″ if no medical care was sought. It is to note that medical care was defined as care from public health facilities/hospitals; private hospitals/clinics/chambers; and NGO health centers/hospitals. On the other hand, care from private pharmacies, unqualified doctors and no care were categorized as no medical care use.

### Independent variables

There are various influencing factors of healthcare utilization which have been categorized as enabling factors, need factors and predisposing factors [25]. We used a modified version of Anderson’s Behavioral Model like other studies [21, 26]. In this study, the predisposing factors were age of children, sex of children, age of mother, mother’s and father’s education level, occupation of mother, number of household members, number of children in the household, household size, birth order, religion, wanted last child, and access of mothers to electronic media. Household wealth index, region of residence, and distance to the nearest health facilty were used as enabling factors in this study. Sex of the child was categorized as male or female, mother’s and father’s education level were categorized as no education, primary education, secondary education and higher education. Mother’s occupation was categorized as no formal occupation/housewife, professionals, working in the agricultural sector and others. Number of children in the household was categorized into three categories as 1–2, 3–5, 6 and above while household size was categorized as 1–4, 5–6 and above 6. Religion was categorized as Muslim and non-Muslim.

There was no data on household income in the BDHS 2014 survey. However, data on household wealth status, computed from household assets and household characteristics, were available. Using Principal Component Analysis (PCA), wealth index was calculated based on the household assets and household characteristics. Availability of radio, television sets, mobile phones, refrigerators, land ownership, livestock ownership etc. were some of the variables which had been used for measuring household assets. Furthermore, characteristics like sources of drinking water, water for cooking and washing, type of fuel for cooking, and type of toilet facilities were also used. Considering the wealth status, households were categorized into five groups such as poorest, poorer, middle, richer, and richest. The wealth index used in this study is a measure which has been used in other DHS and many country-level surveys to measure inequalities [24].

Access of mothers to electronic media (television set/radio) was categorized as “1” if access and “0” if no access considering that particular household has radio/television.

Region of residence had been categorized as urban and rural. Furthermore, region of residence was categorized as seven administrative divisions (Barisal, Chittagong, Dhaka, Khulna, Rajshahi, Rangpur and Sylhet). Distance to the nearest health facility was categorized as health facility within the community, health facility within 1–3 km/s, and health facility with distance above 3 km.

### Statistical analysis

The study used Stata 14.00 software for carrying out all the statistical analyses. Descriptive bivariate analysis was performed using cross tables and chi-square test to investigate the association between the selected variables and use of medical treatment. In the bivariate analysis, those factors having *P*-values less than 5% were chosen in the multivariate analysis. Multivariate logistic regression analysis was employed to capture the association between dependent and independent variables and statistical significance was determined considering the *p*-values. For all models, adjusted odds ratios (AOR) and 95% confidence intervals were reported. Variables with *p* values less than 5% were considered as significant predictors.

## Results

### Prevalence and treatment for childhood illnesses

A total of 7256 mothers who had children aged under 5 years were included in the study. Among them, 3179 mothers (43.81%) reported that at least 1 of their children had suffered from fever/cough within 2 weeks preceding the survey and 370 mothers (5.11%) reported that at least 1 of their children had diarrhea within 2 weeks before the survey. Out of the 3179 children with fever and/or cough, about 37% sought medical treatment while that figure was about 36% (out of 370 children) for diarrhea.

**Fig. 1:**
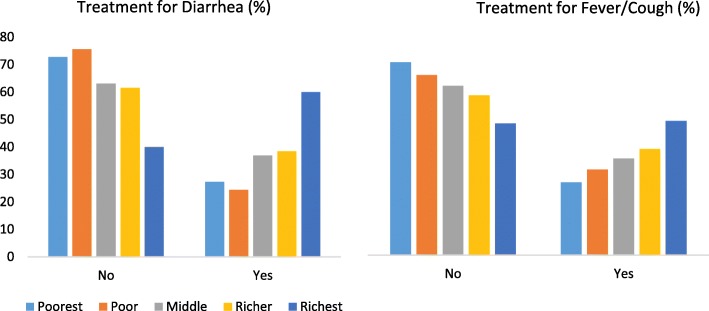
Distribution of wealth indices with corresponding medical care utilization for childhood illnesses in Bangladesh. The figure depicts that as wealth status improved, the rate of utilization of medical care increased for childhood diarrhea and fever/cough and vice-versa

Table 1 shows that the highest use of medical treatment was among the group of children less than 6 months of age (52.92%) while with the increase in age, the utilization of health care decreased. About 37% of the male children who suffered from fever/cough sought medical care while that figure was only 36% for female children. The use of medical treatment among children with fever/cough increased significantly with their mothers’ increased level of education. The likelihood of being taken to any health facility or to any medical provider for treatment increased with households’ improved wealth status. For instance, around 50% of children from the highest wealth quintile were taken to a health facility or a medical provider for treatment of their fever/cough compared to 27.41% of children from the lowest wealth quintile. Figure 1 is also showing that use of medical care for both childhood diarrhea and cough/fever increased with wealth status while the proportion of children receiving no care declined as the wealth status improved.

**Table 1:** Treatment for childhood illnesses according to background characteristics

Variables	Fever/Cough		Diarrhea	
	Treatment, n (%)	Total, n (%)	Treatment, n (%)	Total, n (%)
**Child’s age in months**				
< 6	136(52.92)	257 (100)	08(33.33)	24(100)
6–8	100(44.64)	224(100)	12(46.15)	26(100)
9–11	87(40.85)	213(100)	17(48.57)	35(100)
12–17	152(38.38)	396(100)	33(45.83)	72(100)
18–23	118(36.42)	324(100)	13(30.23)	43(100)
24–35	251(39.10)	642(100)	24(38.71)	62(100)
36–47	179(29.83)	600(100)	19(31.67)	60(100)
48–59	146(27.92)	523(100)	09(18.75)	48(100)
	P < 0.001		*P* = 0.050	
**Gender**				
Female	550(36.50)	1507(100)	62(37.13)	167(100)
Male	619(37.02)	1672(100)	73(35.96)	203(100)
	*P* = 0.759		*P* = 0.817	
**Mother’s age**				
Less than 20	191(37.67)	507(100)	25(39.68)	63(100)
20–34	898(37.15)	2417(100)	105(36.97)	284(100)
Above 34	80(31.37)	255(100)	5(21.74)	23(100)
	*P* = 0.172		*P* = 0.292	
**Mother’s Education**				
No education	155(32.49)	477(100)	17(29.82)	57(100)
Primary	301(32.33)	931(100)	36(29.27)	123(100)
Secondary	546(37.37)	1461(100)	61(39.87)	153,100)
Higher	167(53.87)	310(100)	21(56.76)	37,100)
	P < 0.001		*P* = 0.011	
**Mother’s occupation**				
No formal occupation/ Housewife	904(38.00)	2379(100)	117(39.39)	297(100)
Professionals	61(35.67)	171(100)	9(34.62)	26(100)
Agriculture	122(30.12)	405(100)	4(14.81)	27(100)
Others	80(36.04)	222(100)	5(25.00)	20(100)
	*P* = 0.025		*P* = 0.052	
**No. of children**				
1–2	850(38.43)	2212(100)	103(40.23)	256(100)
3–5	303(34.55)	877(100)	31(29.52)	105(100)
6 or above	16(17.78)	90(100)	1(11.11)	9(100)
	P < 0.001		*P* = 0.044	
**No of children under 5**				
1	809(37.13)	2179(100)	95(39.75)	239(100)
2	296(35.92)	824(100)	32(30.19)	106(100)
3	64(36.36)	176(100)	8(32.00)	25(100)
	*P* = 0.824		*P* = 0.209	
**Place of residence**				
Rural	707(32.30)	2189(100)	75(30.00)	250(100)
Urban	462(46.67)	990(100)	60(50.00)	120(100)
	P < 0.001		P < 0.001	
**Wealth index**				
Poorest	196(27.41)	715(100)	24(27.27)	88(100)
Poor	191(32.21)	593(100)	20(24.39)	82(100)
Middle	236(36.31)	650(100)	24(36.92)	65(100)
Richer	264(39.88)	662(100)	25(38.46)	65(100)
Richest	282(50.45)	559(100)	42(60.00)	70(100)
	P < 0.001		P < 0.001	
**Division**				
Barisal	129 (35.34)	365(100)	18(36.00)	50(100)
Chittagong	239(38.49)	621(100)	35(37.63)	93(100)
Dhaka	188(36.43)	516(100)	25(36.76)	68(100)
Khulna	125(37.20)	336(100)	12(42.86)	28(100)
Rajshahi	142(34.22)	415(100)	14(37.84)	37(100)
Rangpur	153(39.13)	391(100)	7(29.17)	24(100)
Sylhet	193(36.07)	535(100)	24(34.29)	70(100)
	*P* = 0.761		*P* = 0.973	
**Access to electronic media**				
Access	33(60.00)	55(100)	4(44.44)	9(100)
No access	1136(36.36)	3124(100)	131(36.29)	361(100)
	*P* = 0.000		*P* = 0.616	
**Wanted last child**				
Wanted then	665(42.09)	1580(100)	75(40.98)	183(100)
Wanted later	146(36.05)	405(100)	26(42.62)	61(100)
Wanted no more	86(33.46)	257(100)	11(28.21)	39(100)
	*P* = 0.006		*P* = 0.287	
**Birth order**				
First	494(39.58)	1248(100)	69(46.00)	150(100)
Second	517(36.43)	1419(100)	53(31.74)	167(100)
Third	131(34.29)	382(100)	11(28.21)	39(100)
Fourth	27(20.77)	130(100)	2(14.29)	14(100)
	P < 0.001		*P* = 0.009	
**Religion**				
Muslim	1074(36.43)	2948(100)	125(36.66)	341(100)
Non-Muslim	95(41.13)	231(100)	10(34.48)	29(100)
	*P* = 0.154		*P* = 0.815	
**Household size**				
1–4	372(36.15)	1029(100)	45(38.79)	116(100)
5–6	396(34.80)	1138(100)	41(33.33)	123(100)
Above 6	401(39.62)	1012(100)	49(37.40)	131(100)
	*P* = 0.060		*P* = 0.656	
**Father’s highest education**				
No education	230(29.34)	784(100)	28(29.17)	96(100)
Primary	337(33.60)	1003(100)	36(30.25)	119(100)
Secondary	394(40.25)	979(100)	40(37.74)	106(100)
Higher	207(50.24)	412(100)	31(63.27)	49(100)
	P < 0.001		P < 0.001	
**Distance of the nearest health facility**				
Within community	1046(37.32)	2803 (100)	115(38.08)	302(100)
1–3	121(32.88)	368(100)	18(28.57)	63(100)
4 and above	2(25)	8(100)	2(40)	5(100)
	*P* = 0.199		*P* = 0.357

About 38% children of mother who stayed at home sought medical treatment while the use of treatment was low (only 30.12%) for children whose mother were working in the agricultural sector. Table 2 shows that children living in urban areas and children in Rangpur division were more likely to receive this type of treatment than children from rural areas and other divisions. About 60% of children of mother with access to electronic media received treatment while about 36% children of mother with no access to electronic media received treatment. Higher educational status of father increased the likelihood of their children’s use of medical treatment. High birth order of children reduced the likelihood of use of treatment while suffering from fever/cough.

**Table 2:** Multivariate logistic regression result on the association of predictor and outcome variables

Predictor variables	Adjusted Odds Ratio-AOR (95% CI)	
	Treatment for Fever/Cough	Treatment for Diarrhea
**Child’s age in months**		
< 6	2.777 [2.012–3.831]**	2.29 [0.68–7.72]
6–8	1.992 [1.425–2.785]**	3.56 [1.09–11.58]*
9–11	1.654 [1.172–2.334]**	4.96 [1.66–14.77]**
12–17	1.550 [1.161–2.070]**	3.43 [1.33–8.84]*
18–23	1.421 [1.047–1.927]*	1.89 [0.64–5.23]
24–35	1.586 [1.229–2.048]**	2.56 [0.97–6.72]
36–47	1.083 [0.830–1.412]	2.24 [0.82–6.10]
48–59 (ref)	1	1
**Mother’s education**		
No education (ref)	1	1
Primary	0.873 [0.678–1.125]	0.625 [0.28–1.37]
Secondary	0.882 [0.679–1.147]	0.81 [0.36–1.88]
Higher	1.267 [0.868–1.852]	0.55 [0.15–1.98]
**Mother’s occupation**		
No formal occupation (ref)	1	1
Professionals	0.811 [0.574–1 .146]	0.56 [0.21–1.51]
Agriculture	0.967 [0.761–1.230]	0.33 [0.10–1.05]
Others	0.966 [0.718–1.299]	0.58 [0.19–1.73]
**Place of residence**		
Rural (ref)	1	1
Urban	1.559 [1.305–1.862]**	1.460 [0.810–2.634]
**Wealth index**		
Poorest (ref)	1	1
Poor	1.150 [0.898–1.473]	0.70 [0.33–1.48]
Middle	1.290 [1.008–1.651]*	1.12 [0.53–2.37]
Richer	1.246 [0.961–1.615]	1.27 [0.59–2.71]
Richest	1.555 [1.157–2.089]	2.32 [1.04–5.24]*
**Access to electronic media**		
Access (ref)	2.075 [1.181–3.646]*	0.75 [0.18–3.28]
No access	1	1
**Number of children in the household**		
1–2	2.14 [1.19–3.84]*	3.37 [0.38–30.58]
3–5	2.28 [1.27–4.10]**	2.84 [0.31–25.71]
6 or above (ref)	1	1
**Household size**		
1–4	0.904 [0.744–1.010]	0.70 [0.38–1.34]
5–6	0.853 [0.706–1.030]	0.76 [0.41–1.39]
Above 6 (ref)	1	1
**Father’s highest education**		
No education (ref)	1	1
Primary	1.094 [0.881–1.357]	1.005 [0.54–2.01]
Secondary	1.224 [0.966–1.551]	1.098 [0.53–2.29]
Higher	1.425 [1.033–1.967]*	3.060 [1.05–9.03]*

** p < 0.01; * p < 0.05

Children in the age group 9–11 months had higher likelihood of receiving treatment for diarrhea compared to children from other age groups. About 37% of female children received treatment for diarrhea while most of the male children did not receive any treatment (64.04%). Children of mothers with higher education level had greater likelihood of receiving treatment for diarrhea compared to children of mother with no formal education (56.76% versus 29.82%). About 60% of children from the highest wealth quintile received treatment for diarrhea compared to 27.27% of children from the lowest wealth quintile. Table 1 also shows that children living in urban areas were more likely to receive treatment (50%) compared to children living in rural areas (30%). Treatment of children with diarrhea ranged from 29% among children in Rangpur to 42.6% in Khulna. Fewer number of children in the household increased the likelihood of receiving treatment for diarrhea. Mothers’ access to electronic media improved the likelihood of receiving treatment in case of diarrhea. Among children who lived in communities where there are available health facilities within the communities, about 37% of the children received medical treatment for fever and 38% received treatment for diarrhea. On the other hand, children who lived in communities where the distance to the nearest health facilities were 4 km and above, the utilization of medical care for fever/cough was 25% and 40% for diarrhea.

In the bivariate analysis, we found child’s age, mother’s education, mother’s occupation, number of children in the household, place of residence, wealth index, access to electronic media, wanted last children, birth order, and father’s education as important predictors of medical care utilization for childhood illnesses in Bangladesh.

### Determinants of medical treatment seeking behavior for childhood fever/cough

Table 2 shows that age of children, household wealth status, place of residence, household size, father’s education, and access of mothers to electronic media were the significant predictors of the medical treatment seeking behavior for childhood fever/cough. Children aged less than 6 months (AOR = 2.77, *p* < 0.001), 6–11 months (AOR = 1.99, p < 0.001), 9–11 months (AOR = 1.65, p < 0.001), 12–17 months (AOR = 1.55, p < 0.001) and 24–35 months (AOR = 1.58, p < 0.001) had higher likelihood of receiving medical treatment compared to children aged 48–59 months. Besides, children living in urban areas had significantly higher odds of receiving health care from medically trained providers (AOR = 1.56, p < 0.001) than children from rural areas. Compared to children from the poorest households, respondents from the richest and middle wealth status households had significantly higher odds (AOR = 1.55 with *p* < 0.01 and AOR = 1.29 with *p* < 0.05 respectively) of medical care seeking behavior. Children of fathers with higher education level had 1.43 times higher odds of receiving medical treatment than children whose fathers did not receive any formal education and the result was significant at 5% significance level. Number of children in the household was a significant predictor of treatment for children with fever/cough. Children from households with 1–2 children had AOR = 2.14 (*p* < 0.05) of receiving medical treatment compared to children from households with 6 and above children. The presence of television sets or radio (electronic media) in households significantly increased the likelihood of children receiving health care (AOR = 2.08, *p* < 0.05) compared to children of mother with no access to electronic media. Though mothers’ education, occupation, number of under-five children in the household were found as significant predictors of medical care utilization for fever/cough in bivariate analysis, they were not significantly related in the multivariate analysis.

### Determinants of medical treatment seeking behavior for childhood diarrhea

In case of treatment for diarrhea, age of children, households’ wealth status, and fathers’ education level, were found as significant determinants. Children in the age group 6–8 months (AOR = 3.56, *p* < 0.05), 9–11 months (AOR = 4.96, *p* < 0.01) and 12–17 months (AOR = 3.43, *p* < 0.05) had higher likelihood of seeking medical treatment compared to children from 48 to 59 months age group. Households’ wealth status was an important predictor of health care utilization for childhood diarrhea. Children from the richest household quintile had significantly greater odds of receiving medical treatment (AOR = 2.32, *p* < 0.05) compared to children from the poorest household quintile. In addition, fathers’ education had a significant impact on medical care utilization for childhood diarrhea. Children of fathers with higher education had higher likelihood of receiving treatment for diarrhea compared to children of fathers with no education. Though number of children in the household, place of residence, birth order was found as significant predictors of treatment for diarrhea in bivariate analysis, they became insignificant in multivariate analysis.

## Discussion

This study aimed at exploring childhood illnesses and inequalities in health care seeking behavior. Alongside this, the study was conducted with the hope of finding out barriers to treatment for diarrhea, fever and cough which are leading causes of under-five mortality. We found that about 43.81% of the children suffered from fever/cough and 5.11% of the children suffered from diarrhea within 2 weeks preceding the survey. However, only 37% of those suffered from fever/cough sought medical treatment and approximately 36% of those suffered from diarrhea sought medical treatment.

These lower rates of health care services utilization might be a result of self-treatment or medicines from pharmacy that has been well documented in other literature [4, 27–29]. It is to note that this analysis considered only medical treatment. Therefore, it is likely that these percentages of medical care use may be an underestimate for both fever/cough and diarrhea.

Most importantly, this study identified significant factors that led to inequalities in medical care utilization for diarrhea, fever, and/or cough of children less than 5 years of age in Bangladesh. Age of children, household wealth status, parent’s education level, region of residence, number of children in the household, access to electronic media were identified as factors contributing to inequality in health care utilization for childhood illnesses. Our study found age of children as a significant predictor of health care use for childhood diarrhea and fever/cough. Other studies in Bangladesh, Ethiopia, Zambia and Nigeria also found that children in the lower age group were more likely to receive treatment [16, 19, 20, 30]. This pattern may be due to the fact that parents usually perceive their children at the early stage as being especially “delicate” [30]. Wealth status of household was a significant factor of medical treatment for childhood diarrhea, fever and cough. Children from the richest household had higher likelihood of receiving treatment compared to children from poorest households. This finding aligns with findings from other studies in different settings [14, 16, 18–21]. This inequality, largely due to the wealth status of households, can be explained as wealth has a direct positive influence on the utilization of health care services and a lack of resources can create bottlenecks to accessing health care services for children [21]. Paternal educational attainment was significantly associated with inequalities in medical treatment use for children with fever, cough and diarrhea. This finding aligns with our expectation that educated parents are more likely to seek health care for their children and this result is consistent with findings from other studies [18, 20, 21] . The reasoning behind this might be the fact that educated fathers have better knowledge and understanding about childhood illnesses which motivates their spouses to seek health care for their children [20]. In addition, education leads to an increased level of awareness regarding illnesses, and availability of services [21]. We found a significant impact of mothers’ access to electronic media, such as television and radio, on treatment seeking behavior for their children. Other studies conducted in Zambia and Nigeria also found that mothers who had access to electronic media had a greater likelihood of seeking medical treatment for their children [14, 16]. This might be attributed to the fact that information about coughing on television or radio could increase awareness among people about the necessity of seeking health care services for their children timely [21]. We also found that number of children in the household is an important determining factor of using medical care for fever and cough. This finding is consistent with the findings from a study in Tanzania which showed that children from households with fewer number of children are more likely to have better health care than children from households with many children [26]. This study in Tanzania also explained that by the resources and time constraints associated with caring for many young children can create impediments in seeking care timely [31]. Our study further indicated that region of residence was a significant influential factor for inequalities in health care utilization for childhood fever and cough. Children from urban areas are more likely to use medical care compared to children from rural areas. Another study in Bangladesh found similar result that children from rural areas are more likely to use care from traditional providers [18]. This can be attributed to the fact that mothers from rural areas are less educated and they are not aware of where to seek medical care when their children get sick. Furthermore, the majority of the poor people live in rural areas of Bangladesh [24] which might be responsible for the low utilization of medical care among children in rural areas. Though a study in Tanzania found distance to health facilities as an important predictor of medical care utilization [22], we did not find any significant impact of distance on medical care utilization.

As this study is based on a nationally representative household survey data, we can generalize the findings at the national level. However, our study has several limitations. As the survey was cross-sectional in nature, it was not possible to capture any causal association between childhood illnesses and the explanatory variables. Furthermore, all information on childhood illnesses was collected from mothers and the symptoms were not validated by any further medical examination, which might have created recall bias to some extent. Another limitation of this study is that there was no information on the costs of treatment (costs for consultation, diagnostics, medicines, transportation cost, etc.) for childhood illnesses in BDHS data. Finally, mothers could have misconception about childhood illnesses which might lead to misconception about treatment for childhood illnesses too. For instance, it is likely that mothers, in some cases, could not recognize the symptoms of varying types of diseases children suffered from and even if recognized, they could not resort to proper treatment sources.

## Conclusions

The findings of this study show the importance of raising awareness of childhood illnesses in Bangladesh and developing interventions that target children at-risk groups, such as those who live in rural areas, who are from households with less wealth, whose fathers have limited schooling, whose family have many children, whose mothers do not have access to television and radio. Future policies aimed at tackling childhood morbidity and mortality should include comprehensive strategies that impact on socio-economic factors. Since the equitability of access to health care for children is a major concern, interventions should be designed targeting children from households with low socio-economic status. Furthermore, policies for increasing effective awareness raising and health education programs, poverty alleviation programs especially for rural areas can contribute to increased health care utilization for childhood illnesses. If accessibility and affordability of medical care can be increased through working in partnership with public facilities, private facilities as well as community-based organizations, equitable services for childhood illnesses will be boosted.

## Data Availability

The dataset (BDHS 2014) used in this study is publicly available in the DHS website (https://dhsprogram.com/data/dataset/Bangladesh_Standard-DHS_2014.cfm?flag=0).

## Abbreviations

BDHS: Bangladesh Demographic and Health Survey
WHO: World Health Organization
MDG: Millennium Development Goal
SDG: Sustainable Development Goal
ARI: Acute Respiratory Infections
DHS: Demographic and Health Survey
PCA: Principal Component Analysis
AOR: Adjusted Odds Ratio
